# Do you think it's a disease? a survey of medical students

**DOI:** 10.1186/1472-6920-12-19

**Published:** 2012-04-03

**Authors:** Chrissy Erueti, Paul Glasziou, Chris Del Mar, Mieke L van Driel

**Affiliations:** 1 Centre for Research in Evidence-Based Practice, Faculty of Health Sciences and Medicine, Bond University, Gold Coast 4229, Queensland, Australia; 2 Faculty of Health Sciences and Medicine, Bond University, Gold Coast, Queensland, Australia; 3 Discipline of General Practice, School of Medicine, University of Queensland, Brisbane, Australia; 4 Department of General Practice and Primary Health Care, Ghent University, Ghent, Belgium

## Abstract

**Background:**

The management of medical conditions is influenced by whether clinicians regard them as "disease" or "not a disease". The aim of the survey was to determine how medical students classify a range of conditions they might encounter in their professional lives and whether a different name for a condition would influence their decision in the categorisation of the condition as a 'disease' or 'not a disease'.

**Methods:**

We surveyed 3 concurrent years of medical students to classify 36 candidate conditions into "disease" and "non-disease". The conditions were given a 'medical' label and a (lay) label and positioned where possible in alternate columns of the survey.

**Results:**

The response rate was 96% (183 of 190 students attending a lecture): 80% of students concurred on 16 conditions as "disease" (eg *diabetes, tuberculosis*), and 4 as "non-disease" (eg *baldness, menopause, fractured skull *and *heat stroke*). The remaining 16 conditions (with 21-79% agreement) were more contentious (especially *obesity, infertility, hay fever*, alcoholism, and *restless leg syndrome*). Three pairs of conditions had both a more, and a less, medical label: the more medical labels (*myalgic encephalomyelitis, hypertension*, and *erectile dysfunction*) were more frequently classified as 'disease' than the less medical (*chronic fatigue syndrome, high blood pressure*, and *impotence*), respectively, significantly different for the first two pairs.

**Conclusions:**

Some conditions excluded from the classification of "disease" were unexpected (eg *fractured skull *and *heat stroke*). Students were mostly concordant on what conditions should be classified as "disease". They were more likely to classify synonyms as 'disease' if the label was medical. The findings indicate there is still a problem 30 years on in the concept of 'what is a disease'. Our findings suggest that we should be addressing such concepts to medical students.

## Background

Part of medical education consists in learning what is normal and abnormal, and what should and should not be labelled as a disease. The labelling of a condition as a "disease" has important implications for clinicians' communication with patients and their attitudes to management and treatment. Failing to label a condition as a disease may mean effective treatment is not prescribed, while labelling a non-condition as a disease may result in unnecessary treatment. For example, there is an association between the labels used to describe acute respiratory infections by general practitioners (GPs) and their rates of prescribing antibiotics: high prescribers were associated with labels that implied greater seriousness of the condition [1].

Definitions of a disease are vague and circular. The Oxford textbook of Medicine does not define a disease at all, while the Chambers Dictionary defines a disease as "an unhealthy state of body or mind: a disorder, illness or ailment with distinctive symptoms, caused eg by infection" [2]. While labelling of many diseases is straightforward, there are a number of controversial areas. A former editor of the BMJ called for examples of "non-disease" that identified benefits and problems of having a condition labelled "disease". Downsides of labelling include anxiety about prognosis and over-treatment; benefits include enjoying sympathy and better care [2].

Once individuals have been labelled with hypertension, the increase in their absenteeism from work increases by 80% as a direct consequence of the individuals being labelled as 'diseased' [3]. There is disagreement in medicine about what should and should not be labelled "disease". A survey of doctors and medical students 30 years ago found considerable disagreement, particularly for *alcoholism, acne*, and *depression *[4].

Classifications and attitudes change, and there are new candidates for "disease". Medical students are at a stage of their learning where opinions about diseases may be malleable. Accordingly we aimed to survey current medical students' classification of conditions as diseases, using some questions from an original survey for comparison, but including newer candidate conditions [4]. We also tested whether different terms used to describe candidate conditions altered its classification into "disease" or "non-disease".

## Methods

We asked GPs to nominate conditions to be added or removed from the original survey. The new questionnaire was pilot-tested with 12 GPs and medical educators recruited from clinicians and primary care researchers affiliated with the Centre for Research for Evidence Based Practice (CREBP) for research purposes. The survey was modified based on their feedback. Three conditions were deliberately duplicated, using both lay and medical labels to determine if this influenced whether students categorized them as "disease" or "non-disease". The final list contained 36 conditions (Additional file 1).

The survey was completed anonymously and voluntarily in the first fifteen minutes of three lectures conveniently selected for each of three year cohorts. The first three year cohort were selected for the survey based on the educational events timetabled on campus, the structure of the program then has students out on rotation in a variety of hospitals, making accessibility an issue. Students were given fifteen minutes to complete the survey. Approval was granted by the Bond University Human Ethics Committee.

The MBBS (Bachelor of Medicine, Bachelor of Surgery) is based on evidence (Evidence-Based Medicine), and has a strong educational focus on problem-based learning. It is completed in 4 years and 8 months. There is a variety of teaching and learning opportunities provided including case-based small group, small group tutorials, problem based tutorial practical classes, clinical skills and laboratory training, self directed learning, web based, e-learning, interactive seminars and lectures. After 8 blocks, the first "semesters" on campus, students spend 2 full years on clinical rotations.

The year 1 survey was carried out in week 6 of the student commencement of the program, then years 2 and 3. Within the current curriculum there is no specific session on the issue.

For each condition we calculated the percentage of students who categorised it as "disease", with 95% confidence limits. For tests of differences between labels we used a Fisher's exact test (using GraphPad software http://www.graphpad.com/quickcalcs/contingency2.cfm).

## Results

**Figure 1:**
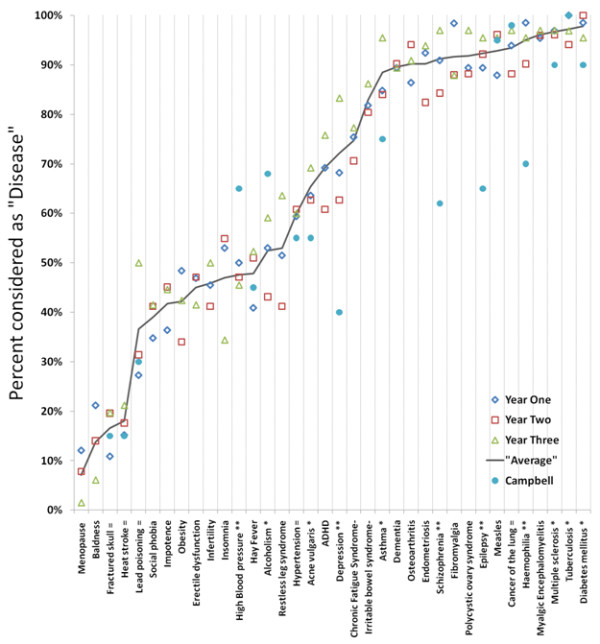
**Percent of students (by course year) classifying conditions as "Disease", compared to Campbell et al 1979**.

We surveyed 190 medical students who attended the lectures from Years 1 (n = 66), 2 (n = 54) and 3 (n = 70) in the School of Medicine at Bond University. The response rates were high: Year 1 (n = 66/66), Year 2 (n = 51/54) and Year 3 (n = 66/70), although only 74% of those enrolled in the medical programme attended the relevant lecture. Of those enrolled in the 3 years of the medical program, 49% were female and the average age was 21, 24, and 26 years for Years 1, 2, and 3 respectively (Figure 1).

There was considerable variation in the proportion classifying conditions as "diseases". *Diabetes *and *tuberculosis *ranked highest with almost all students classing both of these conditions as *diseases*. Lowest ranked were *baldness *and *menopause*, with less than 20% of students labelling these as *diseases*. For several conditions there was a close to even split across students, e.g., *obesity, infertility, hay fever, alcoholism*, and *restless leg syndrome*.

The three pairs of conditions for which we had provided two labels (a more, and a less, medical label) appeared to influence the classification. More medical labels (*myalgic encephalomyelitis, hypertension*, and *erectile dysfunction*) were more frequently classified as "disease" than less medical (*chronic fatigue syndrome, high blood pressure*, and *impotence*), respectively, significantly for the first two pairs, (Table 1).

**Table 1:** Percent classified as disease (pooled across years) for 3 conditions with duplicate names

Condition 1	Number of students labelling condition as *disease *(%)	Condition 2	Number of students labelling condition as *disease *(%)	Percentage difference, % (p-value)
*Myalgic Encephalomyelitis*	174/181 (96)	*Chronic Fatigue Syndrome*	136/182 (75)	21% (< 0.001)
*Hypertension*	108/180 (60)	*High Blood* *Pressure*	87/183 (48)	12% (0.02)
*Erectile dysfunction*	81/180 (45)	*Impotence*	76/182 (42)	3% (NS*)

*p > 0.05

There were only minimal differences in diseases classification across the year cohorts: a mean 65% for Year 1; 64% for Year 2; and 68% for Year 3. The largest apparent difference between years was for *lead poisoning*, which was labelled "disease" by 27% in Year 1 and 50% in Year 3.

## Discussion

We found that students' classification of conditions that they are likely to encounter during their professional lives as "disease", or not, was concordant > 80% across year cohorts for 16 conditions, each of which might be encountered during the first part of a medical programme. Similarly there was concordance < 20% about which conditions classified as "non-disease" for four - *baldness*, *menopause*, *fractured skull *and *heat stroke*. To determine what showed 'good' concordance and 'poor' concordance, there is no universally agreed definition of "good agreement". However, one standard statistical text suggests that for kappa "good agreement" is 0.60 to 0.80 and this seems a reasonable pair of arbitrary cut-points to adopt [5].

The 16 conditions with poor concordance (concordance between 21 and 79%) are of interest. They suggest that the criteria for classifying conditions as "disease" are unclear. For each, one could imagine arguments mounted both for and against. The decision to label something as "disease" clearly has health implications for individuals, but could alternatively be thought of as a societal problem with causes (related to urban design, the economics of food availability and so on) remote from any direct clinical influence.

Some conditions are clearly controversial, such as *chronic fatigue syndrome *and *attention deficit hyperactivity disorder*. Clinicians' opinions are divided on whether these conditions have an organic cause or are linked to personality, or a societal aetiology and our students' ratings may be a reflection of this. A study of 1250 US Healthcare providers showed this with 20% agreeing that "Chronic fatigue syndrome is only in the patients head", with 51% disagreeing but a further 29% answering they did not know [6]. A qualitative study of Australian General Practitioners attitudes and practices to attention deficit hyperactivity disorder showed that those that participated in the focus groups emphasised social, family and environmental factors, however the evidence emphasises the neurobiological nature of attention deficit hyperactivity disorder [7].

The prescription of drugs for attention deficit hyperactivity disorder is managed several times more often in some parts of Australia compared to others, suggests that such controversial classifications can contribute to differences in clinical management [8]. Labelling a condition has implications for the way it is managed. For example, having *bronchitis *justifies the need for antibiotics, whereas the same clinical presentation described as "*a flu" *does not [9]. Even for more 'objective' conditions there may be disagreement. For example, there is confusion on how to treat ductal carcinoma in situ. Women may be given conflicting information on whether to treat or not based on the labelling of this condition. At a recent conference in an audience of 100 surgeons and oncologists, the audience was asked to vote on how they might describe ductal carcinoma in situ to a patient, 50% voted "might become breast cancer," 25% voted "breast cancer at an early stage," and 25% voted "breast cancer and can't be left" [10].

Several previous studies have looked at agreement about labelling and the impact of a medical versus a non- medical label. Participants were psychology and medical students. Both studies resulted in participants considering the medical label more serious, less common and therefore more 'disease-like' than when the condition had the non medical label [11,12].

Medical labels were classified as "disease" more than their lay descriptions in two of three duplicate candidate conditions. There are several possible reasons. Assuming students did not appreciate the conditions were synonyms, they may have been unfamiliar with the more technical description (although for this we might have expected concordance to have increased with the more senior students). Perhaps students simply worked quickly through the list using a heuristic which classified anything technical as *disease *and anything in common language as "*non-disease"*. This might explain the otherwise non-intuitive classifications of *lead poisoning, heat stroke*, and *fractured skull *as "non-disease".

As can be seen from Figure 1, there was little difference between years of students, but some differences when compared to the earlier Campbell survey of secondary school students. For the 19 conditions the surveys had in common, there was no statistically significant difference for 8. Of the 11 where there was a statistically significant difference, the differences were mostly small except for four conditions: *depression, schizophrenia, epilepsy*, and *haemophilia*. While these may reflect societal changes in attitudes, particularly to mental illness, they may also simply reflect sampling differences.

Our methods had shortcomings. The small numbers and multiple comparisons mean that any trends should be interpreted with caution. They may be biased, in that only the years 1, 2 and 3 cohorts were surveyed and only those that attended the lectures. Despite our good response rate, we surveyed students from only one university in Australia who may not be representative of all medical students. There were difficulties in accessing more experienced medical students because they were out on rotations. Surveys are often difficult to interpret, as well as including items from too small a sample of possible conditions. We could look at a different set of conditions in the future as well as a more representative sample of students and practising doctors.

## Conclusions

The data seems to suggest future doctors' attitudes to these conditions is more variable than a naïve approach would assume. As labelling of a condition may influence behaviour and how we manage them, our findings could have implications for the way we teach students about conditions. We may need to review the learning opportunities provided, to allow students to deal with the issues and uncertainties in disease classification. One way could be to review PBL (problem-based learning) cases selected, and ensure ambiguous conditions, such as *chronic fatigue syndrome *or *menopause*, are represented.

Further research into the curriculum and opportunities for student learning including lectures on the defining of a 'disease' and labelling of conditions is required.

## Competing interests

The authors declare that they have no competing interests.

## Authors' contributions

PG and MVD were responsible for study conception and CE designed and wrote the protocol. PG, CDM and CE were primarily responsible for data analysis and interpretation and had full access to all of the data in the study and take responsibility for the integrity of the data and the accuracy of the data analysis. CDM and CE were responsible for acquisition of the data. All authors contributed to the writing of the manuscript. All authors read and approved the final manuscript.

## Pre-publication history

The pre-publication history for this paper can be accessed here:

http://www.biomedcentral.com/1472-6920/12/19/prepub

## Supplementary Material

Additional file 1 Survey - What is a disease? Click here for file
