# Supplementary material for: Do you think it's a disease? a survey of medical students
Source: BMC Med Educ. 2012 Apr 3;12:19. doi: 10.1186/1472-6920-12-19 (PMC3383512; doi:10.1186/1472-6920-12-19)
Supplement: Additional file 1 — Survey - What is a disease? [file 1472-6920-12-19-S1.DOCX]

|  | YES  Disease | NO  Not Disease |  | YES  Disease | NO  Not Disease |
| --- | --- | --- | --- | --- | --- |
| 1. Tuberculosis |  |  | 19. Insomnia |  |  |
| 1. Baldness |  |  | 20. ADHD |  |  |
| 1. Diabetes mellitus |  |  | 21. Hay Fever |  |  |
| 1. Menopause |  |  | 22. Fibromyalgia |  |  |
| 1. Asthma |  |  | 23. Dementia |  |  |
| 1. Obesity |  |  | 24. Osteoarthritis |  |  |
| 1. Infertility |  |  | 25. Impotence |  |  |
| 1. Epilepsy |  |  | 26. Heat stroke |  |  |
| 1. High Blood pressure |  |  | 27. Social phobia |  |  |
| 1. Schizophrenia |  |  | 28. Irritable bowel syndrome |  |  |
| 1. Acne vulgaris |  |  | 29. Endometriosis |  |  |
| 1. Chronic Fatigue Syndrome |  |  | 30. Restless leg syndrome |  |  |
| 1. Depression |  |  | 31. Haemophilia |  |  |
| 1. Lead poisoning |  |  | 32. Polycystic ovary syndrome |  |  |
| 1. Fractured skull |  |  | 33. Erectile dysfunction |  |  |
| 1. Alcoholism |  |  | 34. Measles |  |  |
| 1. Myalgic Encephalomyelitis |  |  | 35. Hypertension |  |  |
| 1. Multiple sclerosis |  |  | 36. Cancer of the lung |  |  |

**What is a disease?**

Professor Paul Glasziou at the Centre for Research in Evidence Based Practice, Bond University, is conducting a survey into how disease is defined because it has implications on diagnosis and prescribing. We would like your opinion on what you would define as a disease.

Should you agree to take part in the survey your participation in no way will impact on the final results of your study. We thank you for your input.
